# 6-Gingerol, a Bioactive Compound of Ginger Attenuates Renal Damage in Streptozotocin-Induced Diabetic Rats by Regulating the Oxidative Stress and Inflammation

**DOI:** 10.3390/pharmaceutics13030317

**Published:** 2021-02-28

**Authors:** Saleh A. Almatroodi, Abdullah M. Alnuqaydan, Ali Yousif Babiker, Mashael Abdullah Almogbel, Amjad Ali Khan, Arshad Husain Rahmani

**Affiliations:** 1 Department of Medical Laboratories, College of Applied Medical Sciences, Qassim University, Buraydah 51452, Saudi Arabia; smtrody@qu.edu.sa (S.A.A.); ababkr@qu.edu.sa (A.Y.B.); mashaelmma@gmail.com (M.A.A.); 2 Department of Medical Biotechnology, College of Applied Medical Sciences, Qassim University, Buraydah 51452, Saudi Arabia; ami.alnuqaydan@qu.edu.sa; 3 Department of Basic Health Sciences, College of Applied Medical Sciences, Qassim University, Buraydah 51452, Saudi Arabia; akhan@qu.edu.sa

**Keywords:** 6-gingerol, diabetes mellitus, inflammation, oxidative stress, renal damage

## Abstract

The aim of present study is to investigate the role of 6-gingerol in ameliorating the renal injury in streptozotocin (STZ)-induced diabetic rats. The diabetes was induced by using a single dose of freshly prepared STZ (55 mg/kg body weight) intraperitoneally which causes the degeneration of pancreatic Langerhans islet β-cells. The diabetic rats were treated with oral gavage of 6-gingerol (10 mg/kg b.w.). The treatment plan was continued for 8 weeks successively and the body weight and fasting blood glucose levels were weekly checked. The biochemical parameters like lipid profile, kidney profile, antioxidant enzyme levels, lipid peroxidation and anti-inflammatory marker levels were investigated after the treatment plant. The pathological condition of kidneys was examined by haematoxylin-eosin (H&E) staining besides this analysis of NF-*κ*B protein expression by immuno-histochemistry was performed. Some of the major parameters in diabetes control vs. normal control were reported as fasting blood glucose (234 ± 10 vs. 102 ± 8 mg/dL), serum creatinine (109.7 ± 7.2 vs. 78.9 ± 4.5 μmol/L) and urea (39.9 ± 1.8 vs. 18.6 mg/dL), lipid profile levels were significantly enhanced in diabetic rats. Moreover, diabetic rats were marked with decreased antioxidant enzyme levels and increased inflammatory markers. Treatment with 6-gingerol significantly restored the fasting blood glucose level, hyperlipidaemia, Malondialdehyde (MDA) and inflammatory marker levels, NF-*κ*B protein expression and augmented the antioxidant enzyme levels in the kidneys of diabetic rats. The kidney damage was significantly normalized by the treatment of 6-gingerol and it provides an evidence that this novel compound plays a significant role in the protection of kidney damage. These findings demonstrate that 6-gingerol reduces lipid parameters, inflammation and oxidative stress in diabetic rats, thereby inhibiting the renal damage. Our results demonstrate that use of 6-gingerol could be a novel therapeutic approach to prevent the kidney damage associated with the diabetes mellitus.

## 1. Introduction

The prevalence of diabetes mellitus is increasing dramatically worldwide and is projected to continue with same pace, if proper guidelines are not taken [1]. At present, there are 382 million people suffering from diabetes and it is presumed that by the end of 2035, this population is may reach up to 592 million, according to the International Diabetes Federation [2].

Diabetic nephropathy, a common late-stage complication of diabetes mellitus patients, usually leads to severe morbidity and mortality. Moreover, this disease is one of the most common microvascular complications of diabetes mellitus [3], and is categorized by persistent proteinuria, advanced loss of renal function and morphological changes, such as mesangial expansion, glomerular hypertrophy, glomerular basement membrane thickening, as well as interstitial fibrosis [4]. A novel analysis has confirmed that early blood glucose control plays a major role in the reduction of the incidence of diabetic nephropathy [5]. Additionally, hyperglycaemic damage to mesangial cells is concerned with the progression of diabetic nephropathy [6,7].

The *β*-cytotoxic drugs such as streptozotocin (STZ) is commonly used to develop diabetes in experimental animals. The STZ induction leads to the necrosis of the pancreatic *β*-cells and also caused various types of pathogenesis [8]. Usually in rats, diabetes is induced by taking 55 mg/kg b.w. of STZ [9]. The STZ is preferred to develop type I diabetic models as it does not lead to neurotoxicity like alloxan. However, the disadvantage of this drug as a single high dose injection leads to lethality.

Numerous drugs such as taurine, melatonin, vitamin C, and vitamin E are used for the treatment and protection of diabetes. Taurine is a ubiquitous sulfur-containing amino acid that is abundant in mammalian tissues. It plays important role in osmoregulation, pharmacological actions and prevention of oxidative tissue damage [10]. Melatonin is a ubiquitous molecule that carries out many functions and it performs different functions as antioxidant and anti-inflammatory activities [11]. Vitamin C (ascorbic acid), plays important role in the inhibition of pathogenesis. This compound protects the cells against lipid peroxidation by scavenging reactive oxygen species (ROS) [12]. Vitamin E, a potent peroxyl radical scavenger, is a chain-breaking antioxidant that prevents the propagation of free radicals [13].

The therapeutic efficiency of such drugs is really limited by some factors such as adverse effects and incapability to regulate blood glucose levels [14,15,16].

In addition, most of the oral hypoglycemic mediators used to improve diabetic nephropathy are adipogenic [17] and synthetic oral hypoglycemic therapies are narrowly linked with numerous toxicities [18].

Therefore, the search for drugs that can protect against kidney damage and limit the side effects of diabetes is needed. In this regard, natural products or their purified compound from medicinal plants play a vital role in the management of diabetes and its associated complications through the regulation of various biological activities. Therefore, some powerful antioxidants present in these medicinal plants could possibly serve as treatments for diabetes related diseases [19].

6-gingerol ((S)-5-hydroxy-1-(4-hydroxy-3methoxyphenol)-3-decan-one) is an aromatic polyphenol, an active ingredient of ginger (*Zingiber officinale*) which belongs to the family *Zingiberaceae*. The role of this compound has been investigated as antioxidant, anti-inflammatory and anti-cancer activities [20,21]. Some studies like antihyperglycemic and antioxidant activities have been investigated earlier [22,23]. Remarkably, the previous studies demonstrate that 6-gingerol shows potent insulin secreting, lipid-lowering, anti-hyperglycemic and antioxidant properties in type-II diabetic animal models [24]. However, some more aspects of its mechanism of action, as anti-glycemic and the reno-protection needs to be examined in detail.

To examine the role of 6-gingerol in the management of diabetic complications, in this study streptozotocin (STZ)-induced renal damage animal models were used to identify some molecular mechanisms through the evaluation of oxidative stress, lipid profile, inflammation and histopathological alterations.

## 2. Materials and Methods

### 2.1. Chemicals

The diabetes inducing drug, Streptozotocin was purchased from Abcam (ab 142155, Cambridge, UK), 6-gingerol (G1045) was purchased Sigma Aldrich, St. Louis, MO, USA. Antioxidant enzymes like superoxide dismutase (SOD) (ab 65354), Glutathione-S-transferase (GST) (ab 65326), catalase (CAT) (ab 83464) and Trichrome Stain Kit (Connective Tissue Stain) (ab150686) were bought from Abcam, Cambridge, UK. ELISA kits for the assay of inflammation by C-reactive protein (ab 108827), TNF-α (ab 46070), IL-1β (ab 100768), and IL-6 (ab 100772) were procured from Abcam, Cambridge, UK. Primary antibodies (TNF-α) and Goat Anti-Mouse IgG H&L (HRP) (abcam, Cambridge, UK) used in this study were purchased from Abcam, Cambridge, UK. All supplementary chemicals used in this study were of high purity grade obtained from commercial sources.

### 2.2. Animals

Healthy male Albino Wistar rats weighing between 180–220 g were procured from the animal house of King Saud University, Riyadh, Saudi Arabia. The animals were kept in polypropylene cages at temperature of 22 ± 2 °C with a relative humidity of 50–60% and a 12 h light/dark cycle in the animal house at College of Applied Medical Sciences, Qassim University. The animal experiments were carried out as per the guidelines of College of Applied Medical Sciences, Qassim University and approved by the Institutional Animal Ethics Committee (2019-2-2-I-5623), College of Applied Medical Sciences, Qassim University. Throughout the experimental duration, the animals consumed a standard pellet diet and water ad libitum.

### 2.3. Experimental Design

The animals were randomly selected and divided into four groups (group I to group IV) with 8 animals in each group (Table 1). All the animals were fasted overnight prior to the start of the experiment. The group I served and normal control and the diabetes was induced in rats (group II, III and IV) by a single intraperitoneal (i.p.) injection of freshly prepared STZ (55 mg/kg b.w.) dissolved in 0.01 M citrate buffer, pH 4.5 [25] except for group I animals which received same buffer as placebo. The blood glucose levels of control and diabetes induced rats were measured through tail vein puncture to check hyperglycaemia. The induction of diabetes was checked after the third day of the STZ injection and animals having fasting blood glucose level more than 200 mg/dL were selected as diabetic and were included for the experiments. Treatments were started on day 4 after STZ injection, and this was considered day 1 for treatment and continued for eight weeks.

### 2.4. Measurement of Body Weight and Biochemical Parameters

Once weekly, the body weights of all the animals in all four groups were recorded to check the change in their overall weight and the results were analysed accordingly. At the end of the study, blood samples were collected and allowed to clot at room temperature for 30 min and serum was obtained by centrifugation at 3.500 rpm for 10 min. The estimation of total cholestrol, triglycerides, and low density lipoprotein were performed by Crescent Diagnostics, Jeddah, KSA and the result were interpreted accordingly. Additionally, creatinine and blood urea were assayed accordingly using reagent kits purchased from Crescent Diagnostics, Jeddah, KSA.

### 2.5. Measurement of Lipid Peroxidation

The lipid peroxidation in the kidney homogenate of experimental group rats was determined by assaying MDA level run in duplicate, and the results obtained were interpreted accordingly.

### 2.6. Determination of Antioxidant Enzymes Levels

Small portion of kidney were harvested from all the rats and stored in phosphate buffer saline. All the tissues were homogenized and centrifuged at 1100× *g* for 15 min at 4 °C. Antioxidant enzymes including SOD, GST, CAT and GSH levels were measured in duplicate, using the available kits, according to manufacturer’s protocol.

### 2.7. Measurement of Pro-Inflammatory Parameters

Inflammatory markers such as CRP, TNF-⍺, IL-6 and IL-1β were measured in duplicate, by using ELISA kits from Abcam Company according to the manufactures protocol and results were interpreted accordingly.

### 2.8. Histopathological Examination of Kidney

All the kidney tissue samples were fixed in neutral formalin and paraffin embedded block was made. After thin sectioning (5 µm), the sections were mounted on slides. The hematoxylin and eosin (H&E) staining was performed to examine the architecture of kidney tissues. Histopathological examination was performed by using a light microscope (Olympus, Tokyo, Japan) and the slide pictures were shot accordingly. The histological findings were performed by double-blinded histopathologist. Furthermore, Masson’s trichrome was used to evaluate the morphology of fibrosis as per the manufacturer’s protocol (Abcam, UK).

### 2.9. Immunohistochemical Evaluation of TNF-alpha

All the kidney tissues were fixed, dehydrated, paraffin embedded, and thin sections were cut. The tissue sections were deparaffinised with xylene, rehydrated with graded alcohol, washed in water and incubated to antigen retrieval in 0.1 M citrate buffer (pH 6.0). After blocking, all the tissue sections were incubated with TNF-⍺ antibody (1:250 dilution) for overnight. After washing with PBS (pH 7.0), the sections were treated with secondary antibody (no dilution as per the manufacturers’ instructions) for 45 min. Finally, these samples were stained with Diaminobenzedine, washed with phosphate buffer salin and mounted.

The stained sections were examined through Olympus (Tokyo, Tokyo, Japan) microscope and the results were interpreted accordingly. The expression pattern of TNF-α was evaluated manually based on the cytoplasmic expression. The quantification of cytoplasmic positivity of TNF-α was evaluated by a pathologist, blinded to the different animal groups. A total of 500 cells from the five different selected regions were counted. The positive stained cells were expressed as the percentage of total cells counted in each case. If less than 5% of cells showed positivity for TNF- α, were considered as negative. If equal or more than 5% of the cells were positive for TNF- α, were considered as positive.

Based on the staining intensity or expression pattern, the stained sections were graded as 0 for no staining/expression, 1 was represented for weak but detectable (~25%) staining, 2 was represented for moderate staining (25–50%) and 3 was represented for intense or high staining (>75%).

### 2.10. Statistical Analysis

All the values are presented as the means ± standard deviation. For comparisons in multiple groups, one-way analysis of variance (ANOVA) was used. The probability, *p*  < 0.05 was considered as statistically significant for a two-tailed test.

## 3. Results

### 3.1. Effect of 6-Gingerol on Body Weight and Blood Glucose Levels

**Figure 1:**
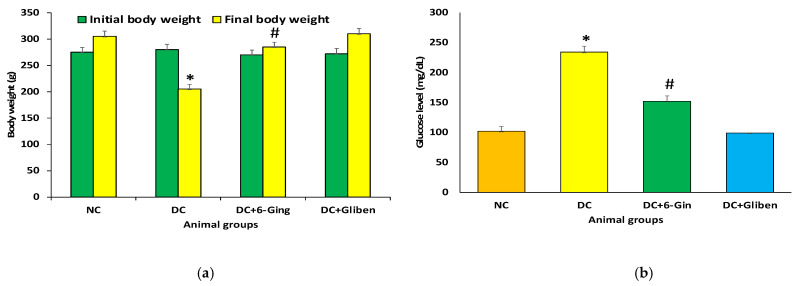
(**a**) The initial and the final body weight of different groups of rats after 8 weeks of experimental design. The rats were equally divided into 4 groups, each group (*n* = 8). Data are represented as mean ± standard error of the mean (SEM). Groups: Normal control (NC); disease control (DC), i.e., STZ-treated group; treatment group (DC + 6-gingerol); positive control (PC) animal treated with STZ + glibenclamide; * *p* < 0.05 (significant difference of final b.w. between DC vs. NC) # *p* < 0.05 (significant difference of final b.w. between DC vs. DC + 6-gingerol). (**b**): The values of serum glucose in different groups of rats after 8 weeks of treatment. The rats were equally divided into 4 groups, each group (*n* = 8). Data are represented as mean ± standard error of the mean (SEM). Groups: Normal control (NC); disease control (DC), i.e., STZ-treated group; treatment group (DC + 6-gingerol); positive control (PC) animal treated with STZ + glibenclamide; * *p* < 0.05 (significant difference of final b.w. between DC vs. NC) # *p* < 0.05 (significant difference of final b.w. between DC vs. DC + 6-gingerol).

The body weight of all experimental rat groups was recorded weekly during the 8-week long experimental period. In comparison with the control rats, there was a significant reduction in the body weight of diabetic rats. After continuous treatment with 6-gingerol to group III animals, this procedure attenuated the weight loss of the diabetic rats. As compared with the diabetes control rats (group II), the surge in body weight was nearly 26.22% in 6-gingerol-treated diabetic rats (Figure 1).

The rats were allowed to fast for 12 h to evaluate their fasting blood glucose. The fasting blood glucose level in group II animals was significantly higher as compared to animals present in control group (*p* < 0.05). As compared to the STZ group, the fasting blood glucose levels in the STZ plus 6-gingerol group were significantly decreased (*p* < 0.05).

### 3.2. Effect of 6-Gingerol on Lipid Profile

**Figure 2:**
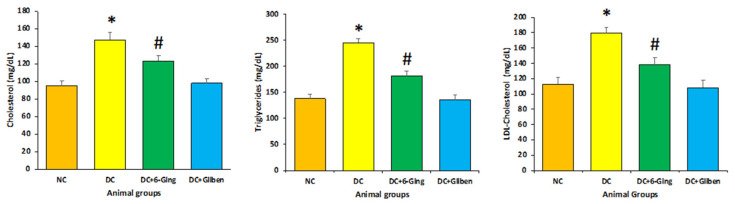
The lipid profile in different groups of rats after 8 weeks of continues treatment. The rats were equally divided into 4 groups, each group (*n* = 8). Data are presented as mean ± standard error of the mean (SEM). Groups: Normal control (NC); disease control (DC), i.e., STZ-treated group; treatment group (DC + 6-gingerol); positive control (PC) animal treated with STZ + glibenclamide; * *p* < 0.05 (significant difference of final b.w. between DC vs. NC) # *p* < 0.05 (significant difference of final b.w. between DC vs. DC + 6-gingerol).

As presented in Figure 2, The STZ-induced diabetic rats had markedly elevated level of TG (238.7 mg/dL vs. 130.9 mg/dL) and TC (147.37 mg/dL vs. 95.34 mg/dL) and LDL-C (179.9 mg/dL vs. 112.8 mg/dL) levels as compared with those of the control group. Animals treated with 6-gingerol (150 mg/kg) showed significantly attenuated the TC (123.45 mg/dL), TG (181.3 mg/dL), LDL-C (138.23 mg/dL) as compared with STZ treated animal group (*p* < 0.05).

### 3.3. Effect of 6-Gingerol on Serum Creatinine, and Blood Urea Level

**Figure 3:**
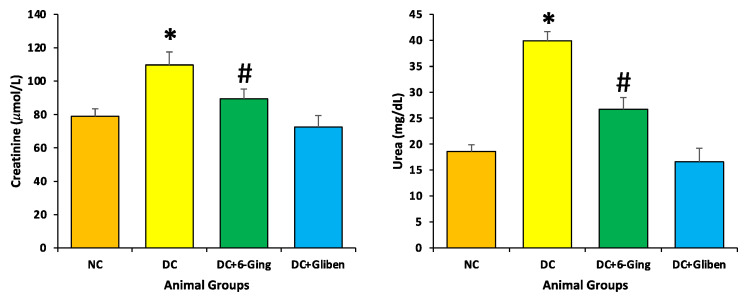
The kidney function test profile (creatinine and urea) in different groups of rats after 8 weeks of treatment. The rats were equally divided into 4 groups, each group (*n* = 8). Data are presented as mean ± standard error of the mean (SEM). Groups: Normal control (NC); disease control (DC), i.e., STZ-treated group; treatment group (DC + 6-gingerol); positive control (PC) animal treated with STZ + glibenclamide; * *p* < 0.05 (significant difference of final b.w. between DC vs. NC) # *p* < 0.05 (significant difference of final b.w. between DC vs. DC + 6-gingerol).

Following the induction of hyperglycemia via STZ, elevated levels of serum creatinine (109.7 µmol/L vs. 78.9 mol/L), and blood urea (39.9 mg/dL vs. 18.6 mg/dL) were observed in the STZ group as compared to the control group. However, 6-gingerol treatment significantly reduced renal dysfunction biomarkers such as serum creatinine (89.39 µmol/L), and blood urea (26.7 mg/dL) as compared to STZ treated animals. These results indicate that 6-gingerol protects rental function in STZ induced diabetes (Figure 3).

### 3.4. Effect of 6-Gingerol on Oxidative Stress

**Figure 4:**
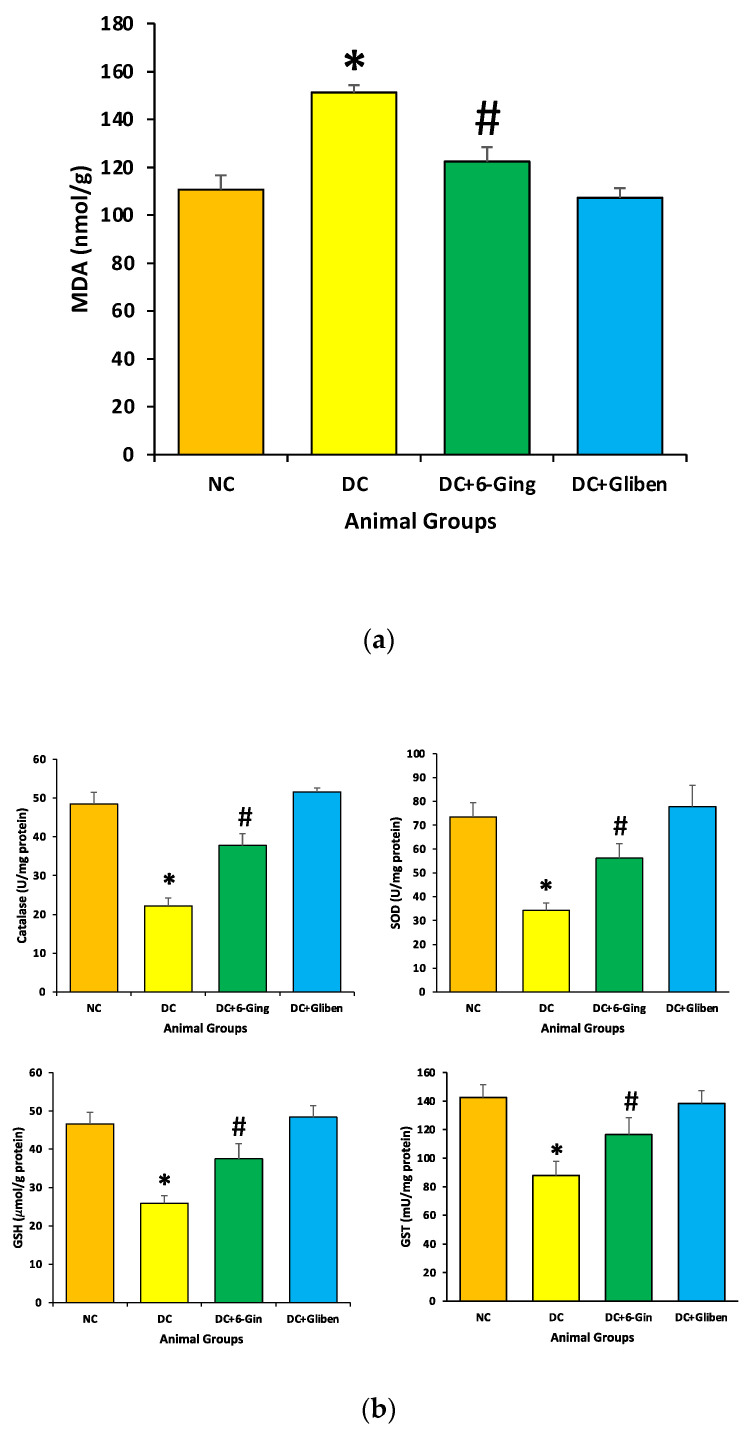
(**a, b**): The lipid peroxidation (MDA) and antioxidant enzyme (CAT, SOD, GST and GSH) level in different groups of rats after 8 weeks of treatment. The rats were equally divided into 4 groups, each group (sample size = 8). Data are shown as mean ± standard error of the mean (SEM). Groups: Normal control (NC); disease control (DC), i.e., STZ-treated group; treatment group (DC + 6-gingerol); positive control (PC) animal treated with STZ + glibenclamide; * *p* < 0.05 (significant difference of final b.w. between DC vs. NC) # *p* < 0.05 (significant difference of final b.w. between DC vs. DC + 6-gingerol).

The protective role of 6-gingerol on the redox status of the normal and diabetic rats was measured via assessment of the levels of MDA, and antioxidants enzyme (CAT, SOD, GST and GSH) levels. STZ-induced diabetic rats showed a significant reduction in CAT (22.2 U/mg vs. 48.5 U/mg), SOD (34.3 U/mg vs. 73.3 U/mg), GST (87.9 U/mg vs. 142.6 U/mg) and GSH (25.9 µmol/g vs. 46.6 µmol/g) level as compared to the control group. Treatment with 6-gingerol meaningfully improved the altered oxidative stress levels in STZ induced diabetic animals resulting in increased antioxidant levels (Figure 4). Moreover, STZ-induced diabetic rats showed a significant surge in kidney MDA levels (151.3 nmol/g vs. 110.7 nmol/g) in comparison with the normal control group. Treatment with 6-gingerol significantly decreased MDA levels in the kidney of diabetic rats.

### 3.5. 6-Gingerol Decrease the Levels of CRP, IL-6, IL-1β and TNF-α

**Figure 5:**
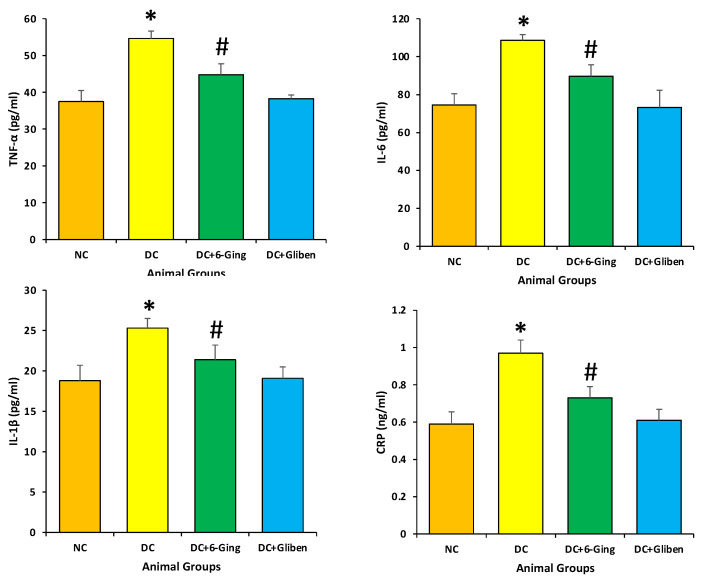
The inflammatory markers level in different groups of rats after 8 weeks of treatment. The rats were equally divided into 4 groups, each group (sample size = 8). Data are summarized as mean ± standard error of the mean (SEM). Groups: Normal control (NC); disease control (DC), i.e., STZ-treated group; treatment group (DC + 6-gingerol); positive control (PC) animal treated with STZ + glibenclamide; * *p* < 0.05 (significant difference of final b.w. between DC vs. NC) # *p* < 0.05 (significant difference of final b.w. between DC vs. DC + 6-gingerol).

The diabetic rats showed noticeable inflammatory responses in the kidney, were significantly enhanced in the diabetic rats TNF-α (54.64 pg/mL vs. 37.49 pg/mL), IL-6 (108.64 pg/mL vs. 74.49 pg/mL), IL-1β (25.3 pg/mL vs. 18.8 pg/mL), CRP (0.97 ng/mL vs. 0.59 ng/mL) as compared with those of the normal rats (*p* < 0.05). Treatment with 6-gingerol significantly decreased the levels of inflammatory markers in the kidneys of diabetic rats as compared to STZ treated only (Figure 5).

### 3.6. Effect of 6-Gingerol on Kidney Architecture

**Figure 6:**
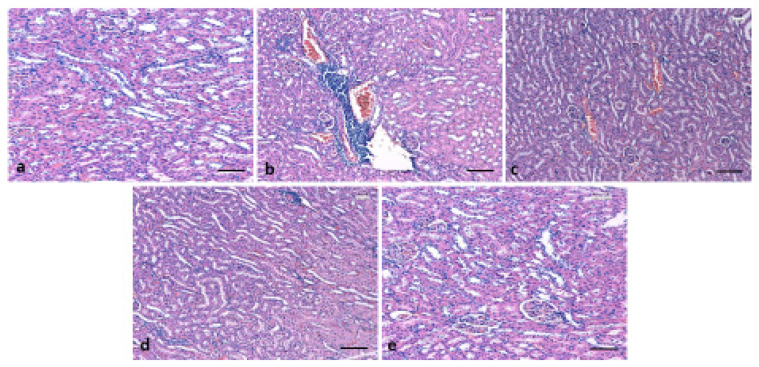
Effect of gingerol in histopathological changes of kidney tissues. (**a**): Typical architecture of kidney in control animals. (**b**): STZ only treated kidney tissues showed infiltration of lymphocytes, thickened glomerular basement membranes and congestion. (**c**): Administration of 6-gingerol markedly mitigated the irregularities in the glomerular and tubular architecture and displayed mild congestion, (**d**): positive control (diabetic rats treated with glibenclamide) showing normal architecture (**e**): Animal group treated with gingerol only showed normal architecture of kidney. (Scale bar = 100 µm).

**Figure 7:**
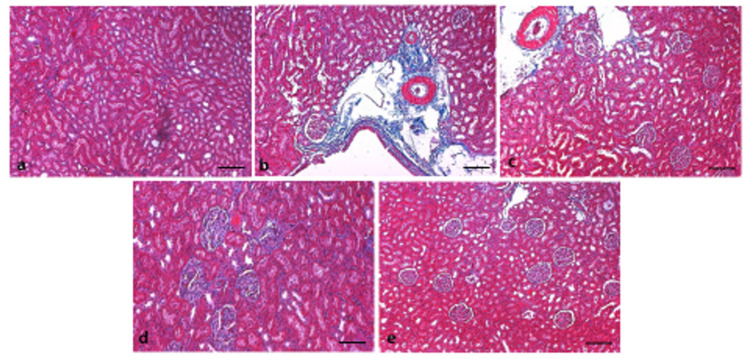
Masson trichrome staining. Masson’s trichrome staining was showing that (**a**): Normal kidney architecture showing no collagen deposition; (**b**): significant increase in collagen deposition in animals treated with STZ was noticed; (**c**): Following treatment with gingerol and STZ treated group collagen deposition was reduced as compared to the disease control group, (**d**): positive control (diabetic rats treated with glibenclamide) not showing any collagen deposition, (**e**): Gingerol-treated group only did not show deposition of collagen. (Scale bar = 100 µm).

The kidney architecture was examined through hematoxylin-eosin (H&E), and Masson’s trichrome staining. Control group rats tissue presented normal cell architecture within the kidney with normal glomeruli and renal tubules. As presented in Figure 6, diabetic group rats displayed infiltration of lymphocytes, thickened glomerular basement membranes, and congestion as compared to normal control rats. However, administration of 6-gingerol for 8 weeks markedly diminished the deformities in both the glomerular and tubular architecture in rats and displayed mild inflammation, and congestion. The kidney damage was significantly normalized by the treatment of 6-gingerol providing evidence that 6-gingerol plays a significant function in the protection of kidney damage. Masson’s trichrome staining was performed on all experimental group tissue to evaluate the distribution of collagen fibers as a previously described method [28]. Control group rats tissue showed normal distribution of collagen. As presented in Figure 7, STZ treated rats displayed high degree of collagen deposition. Treatment with 6-gingerol significantly decreased the collagen deposition when compared with the diabetic control group.

### 3.7. Effects of 6-Gingerol on TNF-α Expression

**Figure 8:**
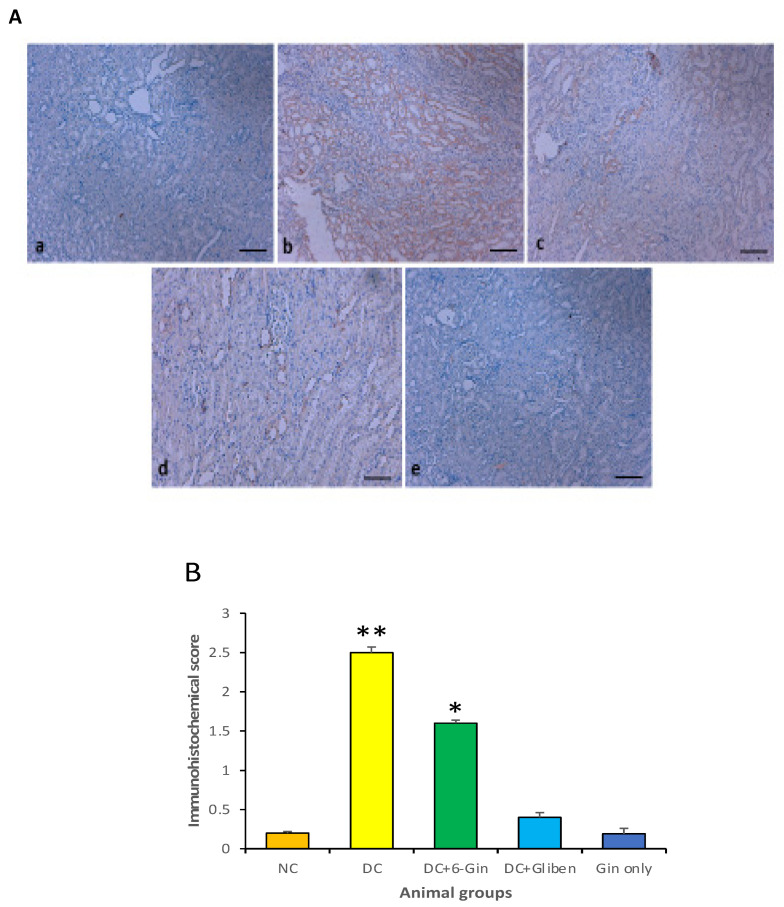
(**A**) (a): The expression of TNF-α protein was not noticed in kidney of normal control group while (b): it showed intense expression in diabetic control group. Moreover, (c): diabetic groups treated with 6-gingerol, exhibited weak expression in diabetic group plus 6-gingerol group. (d): positive control (diabetic rats treated with glibenclamide) not showing any expression, (e): Gingerol-treated group only did not show any expression. (Scale bar = 100 µm). (**B**): represents the graphical representation of TNF-α expression. The expression pattern was high in the diabetic control as compared to the normal control group (** *p* < 0.01). The diabetic group treated with 6-gingerol showed low expression as compared to the diabetic control (* *p* < 0.05). The expression pattern of TNF-α in the positive control and 6-gingerol treated only was statistically insignificant as compared to the control group (*p* > 0.05).

The expression of TNF-α protein was not noticed in kidneys of the control group while it showed intense expression in the diabetic control group as compared to the normal control group (*p* < 0.01). Moreover, diabetic groups treated with 6-gingerol, exhibited low expression in the diabetic group plus the 6-gingerol group as compared to the diabetic control group (*p* < 0.05). As a result of treatment of diabetic rats with 6-gingerol, showed a significant decrease TNF-α protein expression as compared with the diabetic group (Figure 8A,B).

## 4. Discussion

The prevalence of diabetes mellitus has been increasing alarmingly worldwide and is projected to continue in the future at the same pace if proper measuresa are not taken [1]. Animal models are commonly used to evaluate the role of natural compounds in the management of diabetes and its associated complications. STZ is commonly used as diabetes inducing agent in experimental animals due to its capability of specific necrosis induction of pancreatic β-cells that consequences in loss of insulin secretion [29], leading to hyperglycaemia and diabetic complications [30]. A single dose injection of streptozotocin in rats has been reported to caused hyperglycemia as well as renal fibrosis [31].

Here in this study, we appraised the effects of 6-gingerol on STZ-induced renal damage rats through the evaluation of oxidative stress, inflammation, histopathological and biochemical alterations among different treatment groups. 6-gingerol has been found to inhibit the pathogenesis including renal damage through the modulation of various biological activities. It has been identified that gingerol through its antioxidant [32] and anti-inflammatory activities stimulates GLP-1 mediated insulin secretion pathway and upregulates Rab27a/Slp4-a that controls insulin granule exocytosis in pancreatic β-cells. Besides this, it facilitates glucose disposal in skeletal muscle by up-regulating glycogen synthase 1 and increases GLUT4 membrane presentation [20,33]. Gingerol promotes nephroprotective effects by the reduction of oxidative stress, inflammatory processes, and renal dysfunction [34]. In addition, gingerol has been found to down-regulate blood glucose level, creatinine and blood urea nitrogen level. It also regulates pro-inflammatory cytokines, nuclear factor kappa B (N-κB) activation, renal p38MAPK, and TGF-β [35]. Gingerol plays a significant role in improving the condition of renal tissue by alteration in p38MAPK and NF-κB activity, and control inflammatory reaction and oxidative stress [35].

The high level of fasting blood glucose is characterized as diabetes mellitus [35] and is establishes risk factors for coronary heart disease [36].

The fasting blood glucose concentration in the STZ group animals was significantly higher as compared to the control group (*p* < 0.05). In comparison to the STZ group, the fasting blood sugar levels in the STZ plus 6-gingerol group were significantly decreased (*p* < 0.05) (Figure 1b).

The proper insulin level does not only affect the blood glucose level but is also associated with cholesterol regulation including its synthesis and absorption [37]. In the current study, STZ-induced diabetic rats showed a markedly elevated level of TGs, TC and LDL-C levels as compared with those of the control group. Animals treated with 6-gingerol (150 mg/kg) showed significantly attenuated lipid profiles compared with STZ treated animal group (Figure 2). Moreover, 6-gingerol treatment significantly reduced the renal dysfunction biomarkers such as serum creatinine, and blood urea as compared to STZ treated animals. These results demonstrate that 6-gingerol protects rental function in STZ induced diabetes (Figure 3). Some previous reports also support that gingerol significantly down-regulated the blood glucose level, creatinine and blood urea nitrogen level in a dose-dependent fashion [35]. Moreover, gingerol role as antioxidant and anti-inflammatory has been proven [38].

The imbalance between oxidation and anti-oxidant systems, and excessive reactive oxygen species (ROS) generation are the key pathogenic factors in renal disease [39]. It has been explained that hyperglycemia may promote the glomerular mesangial cells and tubular epithelial cells to produce excess ROS, which damage tissue proteins, causes a large number of lipid peroxides and additional worsening of renal oxidative damage [40]. Furthermore, it has been described that hyperglycemia resulted in oxidative injury via shooting up oxidative stress, resulting in overproduction of ROS [41]. Moreover, kidney tissues are more sensitive to ROS linked with oxidative damage because of higher rate of oxygen consumption. In addition, excess ROS could induce an enrichment of MDA content, a good indicator of lipid peroxidation [42]. In the current study, treatment with 6-gingerol appreciably ameliorated the altered oxidative stress levels in STZ induced diabetic animals resulting in increased antioxidant levels. Moreover, STZ-induced diabetic rats showed a significant upsurge in kidney MDA levels. Treatment with 6-gingerol significantly decreased MDA levels in the kidneys of diabetic animals (Figure 4a). The previous study has reported that chronic hyperglycemia resulted in a significant increase in malondialdehyde in the kidney of rats. *Z. officinale* extract efficiently attenuated oxidative stress, and enhanced antioxidant defence in diabetic kidneys [43]. Another study has also reported that diabetic animals treated with gentamicin in an enriched solution of gingerol groups showed amelioration in renal function parameters and reduced lipid peroxidation and nitrosative stress, in addition to an increment in the levels of GSH and SOD activity [34] and 6-gingerol ameliorates gentamicin induced renal cortex oxidative stress [44]

The previous studies based on ginger reported the reno-protective effect through the regulation of lipid peroxidation and maintenance of histopathological changes. The effect of ginger (*Zingiber zerumbet*) exerts an ameliorative effect on renal damage in diabetic rats. Moreover, the histological evaluation demonstrated amelioration of diabetes induced glomerular pathological changes after the treatment with ginger. The finding of this study advocates that the reno-protective effect of *Z. zerumbet* may be similar to the action of metformin and upregulation of the renal nephrin and podocin [45]. The effects of ginger powder on nephropathy induced by diabetes through the measurement of antioxidant capacity and lipid peroxidation were evaluated. The finding of the study revealed that MDL levels in diabetic rats treated with ginger were significantly lower than any other treatment group. This study exhibited that ginger decreases lipid peroxidation and increases in antioxidant capacity and reduction of renal nephropathy [46].

Inflammation plays an important part in the pathogenesis of diabetes [47,48]. Overexpression of inflammatory cytokines provokes the development of diabetes mellitus. In diabetic nephropathy, the production of pro-inflammatory cytokines enhances the changes in glomerular filtration rate and endothelial cell permeability and provokes the production of ROS and other free radicals [49]. Natural products or active compound of medicinal plants shows role in the inhibition of pathogenesis through inhibition of inflammatory process [50,51,52,53]. In the current study, diabetic rats showed significantly increased inflammatory markers, compared with those of the healthy rats. While treatment with 6-gingerol significantly decreased the levels of the inflammatory markers in the kidney of diabetic rats as compared to STZ treated only (Figure 5). Taken together, the above results indicated that 6-gingerol treatment ameliorated diabetes-induced inflammatory reaction in kidneys of diabetic rats. Moreover, a previous study reported that gingerol plays a significant role in improving the condition of renal tissue by alteration in p38MAPK and NF-κB activity, and control inflammatory reaction and oxidative stress [38].

In this study, the histopathological investigation of the sections of kidneys from the STZ treated group of rats showed huge pathological changes such as edema, inflammation, fibrosis, and lymphocytic infiltrate in comparison to the normal structure observed in the normal control group. These findings are in parallel with previous studies and it has been reported that severe destruction as well as glomerular sclerosis occurs in the kidneys of diabetic animals [54]. The administration of 6-gingerol markedly decreased the abnormalities in both the glomerular and tubular architecture in rats and displayed normal renal glomeruli and mild degenerative changes and minimal fibrosis. The kidney damage was significantly normalized by the treatment of 6-gingerol providing evidence that 6-gingerol plays a role in the protection of kidney damage. Additionally, a previous study reported that the treatment with 200 mg/kg of the *Z. officinale* hydroalcoholic extract, the expansion of the mesangial was slightly attenuated and a dose of 400 mg/kg of the same extract ameliorated enlargement of the mesangial in glomeruli [55].

Immunohistochemical study revealed no expression of TNF-α protein in the normal control group while it showed high expression in the diabetic control group. Moreover, diabetic groups treated by 6-gingerol displayed low expression of TNF-α protein. Administration of 6-gingerol markedly decreased the expression levels of TNF-α protein and suggests that it can ameliorate renal damages in diabetic rats, probably through the downregulation of TNF-α protein. A study based on Ursolic acid, a commonly found pentacyclic triterpenoid compound was used to study its effects on some renal disease parameters. The renal structural irregularities and the rise of TNF-α, MCP-1 and IL-1β expression level were diminished by the administration of Ursolic acid [56]. Furthermore, the TUNEL positivity of all treatment groups were examined and no TUNEL positive tissues were seen in any experimental group.

In conclusion, the novelty of our results imply that 6-gingerol attenuated weight loss of diabetic rats and decreased the blood glucose level. Moreover, 6-gingerol shows reno-protective effects in diabetic rats via the regulation of urea and creatinine level, inhibition of oxidative stress, hyperglycemic and inflammatory markers like CRP, IL-6, IL-1β and TNF-α. Moreover, 6-gingerol ameliorates renal fibrosis and pathological changes via the decrease in expression of TNF-α protein in streptozotocin-induced diabetes. The pharmacokinetic and bioavailability studies are required to check its hypoglycemic actions and specific dosage in clinical practice.

## Figures and Tables

**Table 1:** A total of 32 male albino Wistar rats were randomly divided into 4 groups as.

Group Number	Group	Short Name	Treatment Plan
I	Normal Control	NC	Rats were allowed to have free access to a standard pellet diet
II	Diabetes Control	DC	The rats were intraperitoneally injected with freshly prepared STZ (55 mg/kg b.w.) in citrate buffer pH 4.5
III	Diabetes Control + 6-Gingerol	DC + 6-Ging	The diabetic rats were treated with 6-gingerol (10 mg/kg b.w.) [26]
IV	Diabetes Control + Glibenclamide	DC + Gliben	The diabetic rats were treated with Glibenclamide (5 mg/kg b.w.) [27]

## Data Availability

The data used to support the findings of this study are included within the article.
